# Use of Left Gastric Vein as an Alternative for Portal Flow Reconstruction in Liver Transplantation

**DOI:** 10.1155/2016/8289045

**Published:** 2016-08-09

**Authors:** Uirá Fernandes Teixeira, Mayara Christ Machry, Marcos Bertozzi Goldoni, Cristine Kruse, João Alfredo Diedrich, Pablo Duarte Rodrigues, Caroline Becker Giacomazzi, Estéfano Aurélio Negri, Matheus Koop, Carlos Gustavo Spode Gomes, José Artur Sampaio, Paulo Roberto Ott Fontes, Fábio Luiz Waechter

**Affiliations:** ^1^Gastrointestinal and Hepato-Pancreato-Biliary Surgical Division, Universidade Federal de Ciências da Saúde de Porto Alegre and Hospital Santa Casa de Porto Alegre, 90420121 Porto Alegre, RS, Brazil; ^2^Pontifícia Universidade Católica (PUC), Porto Alegre, RS, Brazil

## Abstract

Portal vein thrombosis is observed in up to 10% of liver transplant candidates, hindering execution of the procedure. A dilated gastric vein is an alternative to portal vein reconstruction and decompression of splanchnic bed. We present two cases of patients with portal cavernoma and dilated left gastric vein draining splanchnic bed who underwent liver transplantation. The vein was dissected and sectioned near the cardia; the proximal segment was ligated with suture and the distal segment was anastomosed to the donor portal vein. Gastroportal anastomosis is an excellent option for portal reconstruction in the presence of thrombosis or hypoplasia. It allows an adequate splanchnic drainage and direction of hepatotrophic factors to the graft.

## 1. Introduction

Portal vein thrombosis (PVT) remains a challenge in liver transplantation (LT). It is diagnosed in approximately 10% of patients on waiting list during preoperative evaluation [1, 2]. Portal vein (PV) thrombectomy, use of vascular and artificial grafts, vena cava or renal vein anastomosis, and PV arterialization are among the options for graft revascularization and decompression of splanchnic bed [3, 4]. Although laborious, these techniques have shown encouraging results, making PVT no longer a contraindication for LT and enabling a therapeutic alternative to these patients [5–7]. Its adoption depends on the extent of thrombosis and the surgeon's preference and experience [8]. The use of large collateral varix for portal flow reconstruction in LT is still incipient, with very few clinical reports described. We present our group experience using left gastric vein (LGV) for portal inflow during LT in two patients with PVT.

## 2. Case Report

### 2.1. Case #1

**Figure 1:**
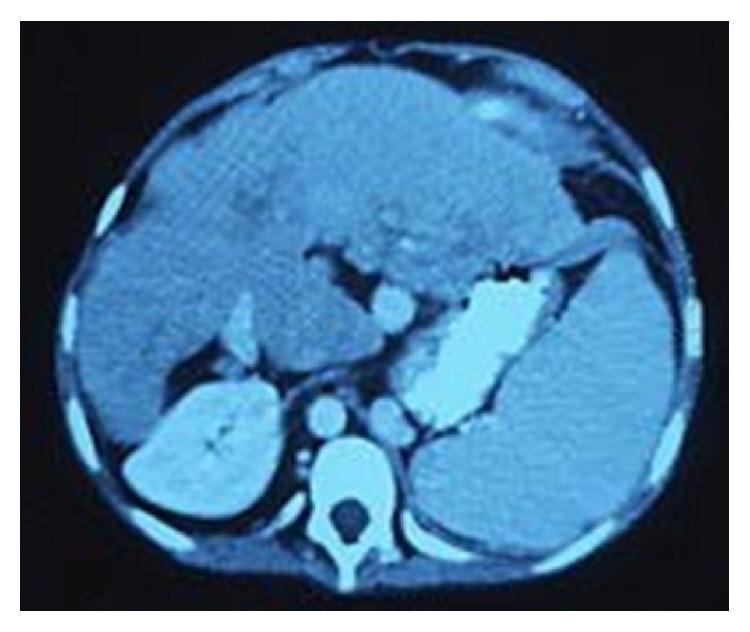
Preoperative angiotomography showing LGV draining splanchnic bed.

A 24-year-old white male presented with primary sclerosing cholangitis associated with splenomegaly and esophageal and gastric varices, with 1 previous episode of bleeding. Preoperative imaging revealed celiac trunk stenosis caused by compression by the median arcuate ligament, associated with portal cavernoma (PC) and dilated LGV draining most of splanchnic flow (Figure 1).

**Figure 2:**
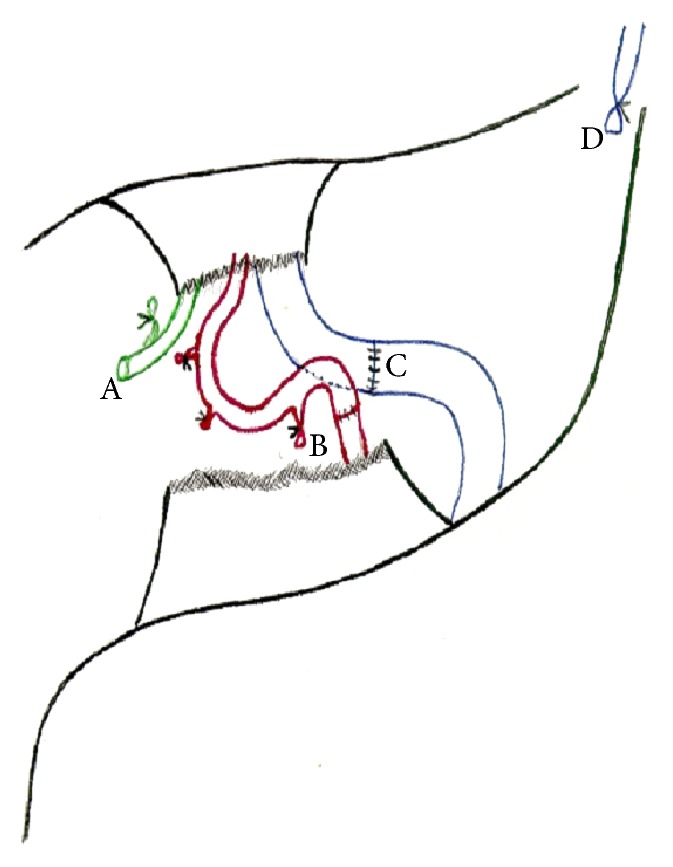
A: donor common bile duct; B: donor-recipient arterial anastomosis; C: gastroportal anastomosis; D: left gastric vein distal neck.

The patient underwent orthotopic LT (OLT) with piggyback reconstruction and surgical division of the median arcuate ligament. PC and dilated LGV with adequate flow were confirmed during operation. LGV was dissected in cranial direction, in order to secure a long graft, and sectioned near the cardia. The proximal neck was ligated with suture and distal segment was rotated laterally to optimize anastomotic openings of donor PV and recipient LGV. An end-to-end anastomosis was performed using polypropylene 5-0 (Figure 2). Intraoperative transfusions were performed with 2 packs of packed red blood cells and 2650 mL of blood from intraoperative cell salvage.

**Figure 3:**
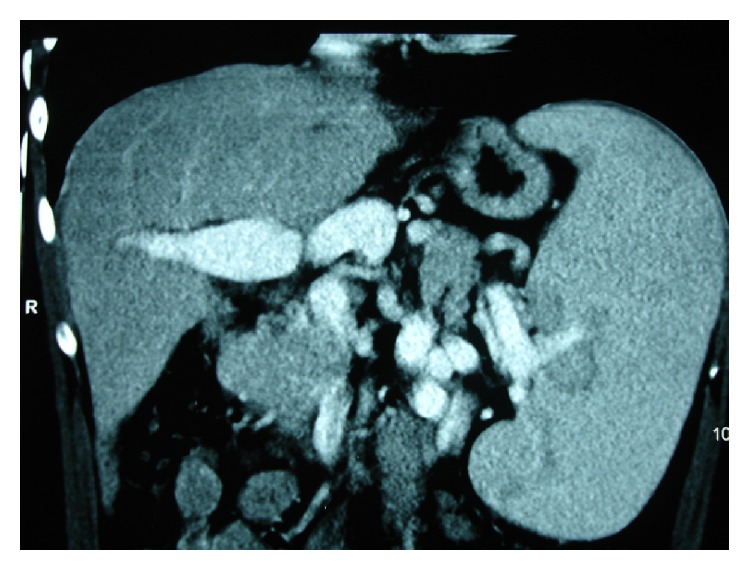
Postoperative angiotomography showing patent gastroportal anastomosis.

Recovery progressed well and uneventful, and the patient was discharged after 11 days with immunosuppressive and antiplatelet medication. Successive Doppler ultrasound studies and angiotomography demonstrated patency of the gastroportal anastomosis and adequate perfusion of the graft in the following 5 years (Figure 3).

### 2.2. Case #2

**Figure 4:**
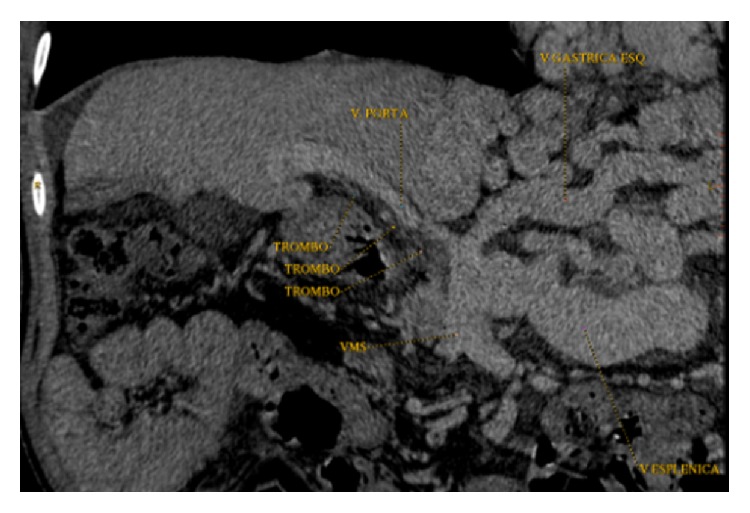
Preoperative computed tomography showing extensive thrombosis of portal and superior mesenteric veins and enlarged left gastric vein draining the bowel.

**Figure 5:**
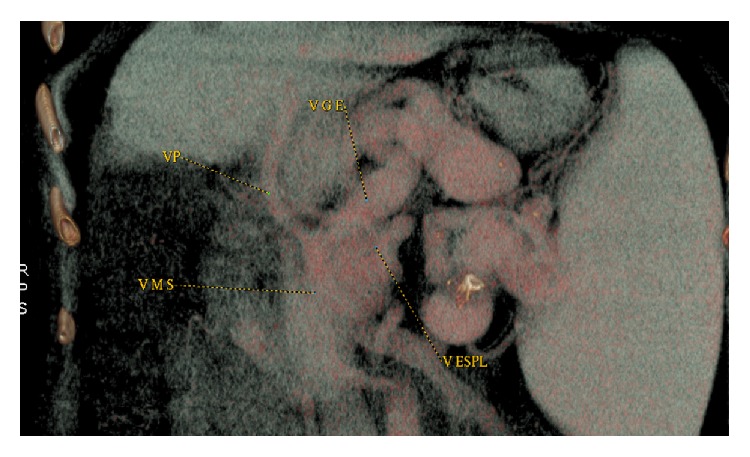
Postoperative angiotomography demonstrating a pervious gastroportal anastomosis.

A 42-year-old white male presented with cirrosis by hepatitis C, previously treated with alpha-interferon. He had diuretic controlled ascitis, previous episodes of esophageal variceal bleeding, and spontaneous bacterial peritonitis. He had undergone resection of a poorly differentiated HCC in liver segment V, measuring 4.6 × 4.5 cm, and chemoembolization of a lesion in liver segment V, measuring 3.5 cm, suggestive of HCC. Child-Turcotte-Pugh score was 10 (grade C) and MELD score was 29. Imaging revealed large esophageal and splenorenal varices, recanalized umbilical vein, chronic splenic vein thrombosis, splenomegaly, patent celiac trunk, PVT extended to proximal SMV, and LGV measuring 2.8 cm (Figure 4). The patient underwent OLT with Belghiti reconstruction. Total PV, splenic vein, and proximal SMV thrombosis and absence of hepatopetal flow were confirmed during operation. Donor PV was anastomosed to recipient LGV in an end-to-end fashion, using polypropylene 5-0 continuous sutures (Figure 5). Bleeding was minimal; no transfusions were performed during the procedure. Postoperative Doppler ultrasonography evidenced patency of the gastroportal anastomosis and hepatopetal flow. Patient recovery progressed well and he was discharged after 15 days with immunosuppressive medication.

## 3. Discussion

Portal vein thrombosis is a complication of end-stage liver disease and may extend to splenic vein and superior and inferior mesenteric veins as well as the splanchnic bed. It is caused by a decrease in portal flow from architectural changes in hepatic parenchyma, periportal lymphangitis and hypercoagulability. Implications of this condition include decrease in liver function and development and aggravation of portal hypertension, due to the reduction in portal flow [9]. Male sex, HCC, cryptogenic cirrhosis, active chronic hepatitis, previous splenectomy, and TIPS are risk factors. Preoperatory imaging exams detect PVT in up to 10% of LT candidates [1, 2]. Recanalized PV and hepatopetal venous flow can occur through vascular neoformation, resulting in a PC. Even though PVT is no longer considered a contraindication in LT [5, 6], it remains a risk factor associated with posttransplant morbidity and mortality, directly affecting graft patency [10–14]. Technical difficulties increase surgery time and lead to severe bleeding; inadequate blood flow may result in graft disfunction or loss and rethrombosis [3, 12, 13, 15–20].

According to PVT extension across splanchnic bed, some technique alternatives to reconstruct PV flow are considered [3, 4, 8]. Extention to proximal SMV with distal patency may be managed with a thrombectomy or mesoportal jumping graft using donor iliac vein [7]. Total compromising of SMV may be managed with portocaval hemitransposition [16] or renal vein anastomosis [17, 21]. Multivisceral transplantation and inclusion of a distal splenorenal shunt come up as an option for diffuse mesenteric and portal vein thrombosis [22–24]. Although well established, these techniques possess significant inconveniences.

Use of mesenteric vein involves a laborious and careful dissection of peripancreatic mesentery root, as well as an extra anastomosis for vascular graft interposition. Portocaval hemitransposition and renal vein anastomosis include dissection of retroperitoneal region with neovascular formations. In addition, portal hypertension is not solved; therefore the risk of gastrointestinal hemorrhage remains and the graft tends to progressive atrophy for lack of hepatotrophic factors [18, 21, 25].

A spontaneous distal splenorenal shunt is an excellent alternative to a portal flow reconstruction, as hepatotrophic factors flow to graft is assured and major dissection of retropancreatic region, associated with severe bleeding, is not required [22]. Distal splenorenal anastomosis requires laborious dissection, results in major blood loss, and may lead to splenectomy [23]. Multivisceral transplantation is associated with higher rates of morbidity and mortality in 1 and 5 years [24], besides the need for a high complex structure and specific trained surgical team [23].

Heterotopic LT is also an option, especially when reconstruction of portal flow using SMV is possible [26]. Nevertheless, the need for intra-abdominal space, high technique complexity, lack of hepatotrophic factors to graft, and requirement of biliary reconstruction are disadvantages. The use of other veins, such as LGV [10, 27], middle colic vein [28], splenomesenteric confluence [29, 30], splanchnic venous confluence [31], and common bile duct drainage vein [32], is also an alternative.

To minimize morbidity, the consensus is reconstruction of portal flow preferably from splanchnic territory, in order to prevent swirling flow, torsion, and stenosis. Recommendations are direct anastomosis to PV trunk and avoidance of jumping grafts and artificial grafts. Anastomotic openings must be adequate to obtain laminar flow [33]. Anastomotic borders must be everted to prevent stenosis. Accessory veins draining part of splanchnic bed must be ligated to impede low flow to portal anastomosis. They usually present as spontaneous splenorenal shunts and dilated LGV.

In our reports, the use of cranially dissected LGV allowed the fulfillment of the cited requirements for a good vascular anastomosis: optimization of anastomotic openings in size and preparation, great mobility of the vascular graft, and exemption from jump grafts and artificial grafts. It also allows splanchnic drainage to graft, securing flow of hepatotrophic factors, and regularizing high preoperative splanchnic blood pressure levels. LGV has few tributaries and it is positioned anteriorly in retroperitoneal region, close to the emergence of splenic artery from the celiac trunk, making its dissection more simple and secure. Yet, manipulation must be cautious, for varicose vessels wall is thin, fragile, and easily torn, particularly during sutures.

Although little experience with LGV as an alternative for portal flow reconstruction in LT is described, no difference in morbidity, mortality, and graft patency is observed. Therefore, this technique presents as an excellent option for portal reconstruction in the presence of thrombosis or hypoplasia.

## References

[1] Manzanet G., Sanjuán F., Orbis P. (2001). Liver transplantation in patients with portal vein thrombosis. *Liver Transplantation*.

[2] Tzakis A. G., Kirkegaard P., Pinna A. D. (1998). Liver transplantation with cavoportal hemitransposition in the presence of diffuse portal vein thrombosis. *Transplantation*.

[3] Rodríguez-Castro K. I., Porte R. J., Nadal E., Germani G., Burra P., Senzolo M. (2012). Management of nonneoplastic portal vein thrombosis in the setting of liver transplantation: a systematic review. *Transplantation*.

[4] Raja K., Jacob M., Asthana S. (2014). Portal vein thrombosis in cirrhosis. *Journal of Clinical and Experimental Hepatology*.

[5] Cho J. Y., Suh K.-S., Shin W. Y., Lee H. W., Yi N.-J., Lee K. U. (2008). Thrombosis confined to the portal vein is not a contraindication for living donor liver transplantation. *World Journal of Surgery*.

[6] Pécora R. A. A., Canedo B. F., Andraus W. (2012). Portal vein thrombosis in liver transplantation. *Arquivos Brasileiros de Cirurgia Digestiva*.

[7] Lai Q., Spoletini G., Pinheiro R. S., Melandro F., Guglielmo N., Lerut J. (2014). From portal to splanchnic venous thrombosis: what surgeons should bear in mind. *World Journal of Hepatology*.

[8] Yerdel M. A., Gunson B., Mirza D. (2000). Portal vein thrombosis in adults undergoing liver transplantation: risk factors, screening, management, and outcome. *Transplantation*.

[9] Carr B. I., Pancoska P., Branch R. A. (2010). Tumor and liver determinants of prognosis in unresectable hepatocellular carcinoma: a large case cohort study. *Hepatology International*.

[10] Stieber A. C., Zetti G., Todo S. (1991). The spectrum of portal vein thrombosis in liver transplantation. *Annals of Surgery*.

[11] Jamieson N. V. (2000). Changing perspectives in portal vein thrombosis and liver transplantation. *Transplantation*.

[12] Ghabril M., Agarwal S., Lacerda M., Chalasani N., Kwo P., Joseph Tector A. (2016). Portal vein thrombosis is a risk factor for poor early outcomes after liver transplantation: analysis of risk factors and outcomes for portal vein thrombosis in waitlisted patients. *Transplantation*.

[13] Qi X., Dai J., Jia J. (2015). Association between portal vein thrombosis and survival of liver transplant recipients: a systematic review and meta-analysis of observational studies. *Journal of Gastrointestinal and Liver Diseases*.

[14] Englesbe M. J., Schaubel D. E., Cai S., Guidinger M. K., Merion R. M. (2010). Portal vein thrombosis and liver transplant survival benefit. *Liver Transplantation*.

[15] Lerut J., Tzakis A. G., Bron K. (1987). Complications of venous reconstruction in human orthotopic liver transplantation. *Annals of Surgery*.

[16] Shaked A., Busuttil R. W. (1991). Liver transplantation in patients with portal vein thrombosis and central portacaval shunts. *Annals of Surgery*.

[17] Liu C., Tsai H.-L., Loong C.-C., Hsia C.-Y., Chin T., Wei C. (2006). ENDO-GIA staplers for side-to-side anastomosis of veins. *European Journal of Vascular and Endovascular Surgery*.

[18] Selvaggi G., Weppler D., Nishida S. (2007). Ten-year experience in porto-caval hemitransposition for liver transplantation in the presence of portal vein thrombosis. *American Journal of Transplantation*.

[19] Emiroglu R., Sevmis S., Moray G., Savas N., Haberal M. (2007). Living-donor liver transplantation: results of a single center. *Transplantation Proceedings*.

[20] Robles R., Fernández J. Á., Hernández Q. (2004). Eversion thromboendovenectomy in organized portal vein thrombosis during liver transplantation. *Clinical Transplantation*.

[21] Lee S.-G., Moon D.-B., Ahn C.-S. (2006). Ligation of left renal vein for large spontaneous splenorenal shunt to prevent portal flow steal in adult living donor liver transplantation. *Transplant International*.

[22] Miyamoto A., Kato T., Dono K. (2003). Living-related liver transplantation with renoportal anastomosis for a patient with large spontaneous splenorenal collateral. *Transplantation*.

[23] Kato T., Levi D. M., Defaria W., Nishida S., Tzakis A. G. (2000). Liver transplantation with renoportal anastomosis after distal splenorenal shunt. *Archives of Surgery*.

[24] Tzakis A. G., Kato T., Levi D. M. (2005). 100 Multivisceral transplants at a single center. *Annals of Surgery*.

[25] Ravaioli M., Zanello M., Grazi G. L. (2011). Portal vein thrombosis and liver transplantation: evolution during 10 years of experience at the university of bologna. *Annals of Surgery*.

[26] Muscari F., Suc B., Fourtanier G., Escat J. (2001). Liver transplantation in patients with a non-functional portal vein: an original technique. *Annales de Chirurgie*.

[27] Lacerda C. M., De Melo P. S. V., Amorim A., Carvalho G., Pereira L. B. (2002). The left gastric vein as an alternative to portal reconstruction in orthotopic liver transplantation. *Transplantation Proceedings*.

[28] Rudroff C., Scheele J. (1998). The middle colic vein: an alternative source of portal inflow in orthotopic liver transplantation complicated by portal vein thrombosis. *Clinical Transplantation*.

[29] Neuhaus P., Bechstein W. O., Blumhardt G., Steffen R. (1990). Management of portal venous thrombosis in hepatic transplant recipients. *Surgery Gynecology and Obstetrics*.

[30] Moreno Gonzalez E., Garcia Garcia I., Gomez Sanz R. (1990). Liver transplantation and portal thrombosis. *Minerva Chirurgica*.

[31] Castaldo P., Langnas A. N., Stratta R. J., Lieberman R. P., Wood R. P., Shaw B. W. (1991). Successful liver transplantation in a patient with a thrombosed portomesenteric system after multiple failed shunts. *American Journal of Gastroenterology*.

[32] Cherqui D., Duvoux C., Rahmouni A. (1993). Orthotopic liver transplantation in the presence of partial or total portal vein thrombosis: problems in diagnosis and management. *World Journal of Surgery*.

[33] Lee S.-G., Hwang S., Kim K.-H. (2003). Approach to anatomic variations of the graft portal vein in right lobe living-donor liver transplantation. *Transplantation*.

